# Identification of the methyltransferase targeting C2499 in *Deinococcus radiodurans* 23S ribosomal RNA

**DOI:** 10.1007/s00792-015-0800-z

**Published:** 2015-11-21

**Authors:** Julie Mundus, Karen Freund Flyvbjerg, Finn Kirpekar

**Affiliations:** Department of Biochemistry and Molecular Biology, University of Southern Denmark, Campusvej 55, 5230 Odense M, Denmark

**Keywords:** Posttranscriptional modification, Methyltransferase, *Deinococcus radioduran*, 23S rRNA, Growth defect

## Abstract

The bacterium *Deinococcus radiodurans*—like all other organisms—introduces nucleotide modifications into its ribosomal RNA. We have previously found that the bacterium contains a Carbon-5 methylation on cytidine 2499 of its 23S ribosomal RNA, which is so far the only modified version of cytidine 2499 reported. Using homology search, we identified the open reading frame *DR_0049* as the primary candidate gene for the methyltransferase that modifies cytidine 2499. Mass spectrometric analysis demonstrated that recombinantly expressed DR0049 protein methylates *E. coli* cytidine 2499 both in vitro and in vivo. We also inactivated the *DR_0049* gene in *D. radiodurans* through insertion of a chloramphenicol resistance cassette. This resulted in complete absence of the cytidine 2499 methylation, which all together demonstrates that *DR_0049* encodes the methyltransferase producing m^5^C2499 in *D. radiodurans* 23S rRNA. Growth experiments disclosed that inactivation of *DR_0049* is associated with a severe growth defect, but available ribosome structures show that cytidine 2499 is positioned very similar in *D. radiodurans* harbouring the modification and *E. coli* without the modification. Hence there is no obvious structure-based explanation for the requirement for the C2499 posttranscriptional modification in *D. radiodurans*.

**Electronic supplementary material:**

The online version of this article (doi:10.1007/s00792-015-0800-z) contains supplementary material, which is available to authorised users.

## Introduction

The ribosome is the ubiquitous protein synthesising machinery in all cells and in organelles like mitochondria and chloroplasts. Overall structural and functional features are remarkably similar in ribosomes independent of phylogenetic origin, exemplified by the structure of the ribosomal RNAs (rRNA) (Letsch et al. 2010; Petrov et al. 2013, 2014) and likely the mechanism of peptide bond formation (Beringer et al. 2005; Ben-Shem et al. 2011). Despite these spatial and functional similarities, the primary structure of rRNA and ribosomal proteins not only varies between organisms, but also the pattern of modifications in these components differs. This is illustrated by the posttranscriptional modifications in rRNA, where distinctive patters of modifications have been identified in essentially all rRNAs examined, [e.g. (Maden 1990; Kowalak et al. 2000; Guymon et al. 2006; Emmerechts et al. 2008; Schnare and Gray 2011; Taoka et al. 2015)]. Some individual rRNA modifications are conserved within a domain of life, or even across domains. For example, psedouridinylation of uridines 1915 and 1917 (*E. coli* numbering used throughout) in 23S rRNA appears nearly universally and are found in bacteria, archaea, invertebrates, vertebrates, plants and organelles (Maden 1990; Ofengand and Bakin 1997; Kirpekar et al. 2005; Mengel-Jorgensen et al. 2006; Blaby et al. 2011; Schnare and Gray 2011); Likewise is ribose methylation of U2552 (Um2552) in 23S rRNA highly conserved (Baer and Dubin 1981; Maden 1988; Lane et al. 1992; Sirum-Connolly et al. 1995; Higa et al. 2002; Kirpekar et al. 2005; Mengel-Jorgensen et al. 2006; Liang et al. 2007). 16S rRNA position 966 constitutes a variation of the theme where the structurally equivalent positions are modified in all domains of life, though the precise nature of the modifications varies (Kowalak et al. 2000; Guymon et al. 2006; Emmerechts et al. 2008).

These highly conserved modifications are generally important for organismal fitness as assayed by inactivation of the genes encoding the enzymatic machinery that introduce the rRNA modifications. The absence of RluD that makes pseudouridine 1911, 1915 and 1917 in *E. coli* 23S rRNA leads to a severe growth defect phenotype with flaws in ribosome assembly (Raychaudhuri et al. 1998; Gutgsell et al. 2005), and effects on translational termination in vitro accompanies lack of these three pseudouridines (Kipper et al. 2011). The 1911, 1915 and 1917 pseudouridines have been shown to play roles in translation, rRNA turnover and ribosome structure in yeast (Liang et al. 2007), which—like all eukaryotes and most archaea—introduces pseudouridinylations using a small nucleolar RNA-based machinery. Um2552 is synthesised in *E. coli* by the heat-shock induced methyltransferase RlmE (previously denoted RrmJ and FtsJ) (Caldas et al. 2000), and an inactive *rlmE* gene is accompanied by slow growth, defects in ribosome assembly and reduced in vitro protein synthesis (Bügl et al. 2000; Caldas et al. 2000). An unusual combination of the RlmE-homologue Sbp1 and the small nucleolar RNA snR52 is responsible for the ribose methylation of U2552 in yeast (Bonnerot et al. 2003), where marked effects on growth and ribosome biogenesis (Bonnerot et al. 2003) as well as in vitro translational fidelity (Baxter-Roshek et al. 2007) are observed when this modification system is non-functional. 16S rRNA  interacts with the first tRNA anticodon nucleotide as revealed by X-ray diffraction ribosome-tRNA co-crystals (Korostelev et al. 2006; Selmer et al. 2006) of *Thermus thermophilus*, from where no functional studies of the modification exist. Knockout of the RsmD methyltransferase that monomethylates G966 in *E. coli* leads to a very modest effect on fitness in co-culturing experiments with the wild-type strain (Lesnyak et al. 2007), but abolishment of the G966 methylation together with the absence of the position-5 methylation on the neighbouring C967 affects both growth and translational initiation (Burakovsky et al. 2012).

From the above examples and others not discussed, it is relatively clear that evolutionary conserved rRNA modifications contribute significantly to the vigour of organisms, which is also what common logic would predict. There are also several examples—mainly from prokaryotes—of species-unique rRNA modifications. U2449 in *E. coli* 23S rRNA is modified to a dihydrouridine (Kowalak et al. 1995), but the modifying enzyme together with the function of the modification remain unidentified. A571 of *Haluarcula marismortui* 23S rRNA is methylated and interacts with nucleotide 2030 (Kirpekar et al. 2005); interestingly, A571 is unmodified in *E. coli* but A2030 is methylated, which suggest a structural significance of the posttranscriptional methyl group in this part of the 23S rRNA. Species-unique modifications can contribute to the phenotypic characteristics of a given species as exemplified in the following: RsmF in *E. coli* methylates 16S rRNA C1407 on cytosine Carbon-5 (Andersen and Douthwaite 2006), and *T. thermophilus* harbours an RsmF with broader specificity in that C1400 and C1404 are also Carbon-5 methylated (Demirci et al. 2010). RsmF in *T. thermophilus* has clear phenotypic impact, in that its deficiency strongly limits bacterial growth at temperatures outside the optimal (Demirci et al. 2010); hence this modification system contributes to the distinctive temperature characteristic of *T. thermophilus*.

**Fig. 1:**
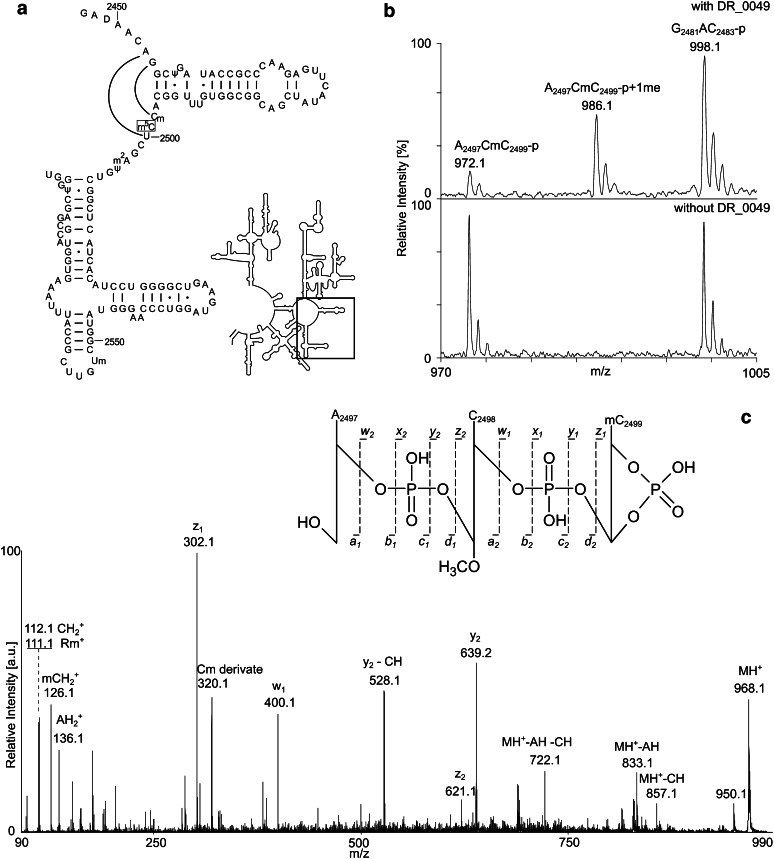
In vivo function of DR0049 on *E. coli* 23S rRNA. **a** Secondary structure of *E. coli* 23S rRNA 3′-end with zoom on the part of the peptidyl transferase centre that harbours C2499. The m^5^C2499 is boxed, and base pairs Cm2498-G2454 and U2500-A2453 observed in the X-ray crystallography structure of the *E. coli* ribosome are indicated. **b** Mass spectrometric identification of DR0049-catalysed methyl group addition to the RNase A generated product A_2497_CmC_2499_-p of 23S rRNA. The product is primarily di-methylated (*m*/*z* 986.1) when *DR_0049* is present (*upper panel*), but exclusively monomethylated (*m*/*z* 972.1) in the original *E. coli* strain (*lower panel*). **c**. Tandem mass spectrometry of a 2′–3′-cyclic phosphate version of the methylated A_2497_CmC_2499_ RNase A product (*m*/*z* 968.1); major backbone fragments are assigned. The masses of the *z*_1_ and *w*_1_ ions reveal that nucleotide C2499 is methylated, while *m*/*z* 126.1 is a signal from a methylated cytosine originating from m^5^C2499; Rm^+^ is a ribose derivative that is diagnostic for 2′-*O*-methylated ribose (from endogenous Cm2498)

*Deinococcus radiodurans* is a species with an extreme resistance to radiation and also desiccation (Mattimore and Battista 1996), and it appears plausible that the bacteria’s protein synthesis is adjusted so the organism can cope with stress conditions. The structure of *D. radiodurans* large ribosomal subunit has been revealed at high resolution (Harms et al. 2001; Schlunzen et al. 2005; Belousoff et al. 2011), which makes the study of rRNA modifications particularly relevant in this organism. We have identified a couple of modified nucleotides in *D. radiodurans* 23S rRNA [(Havelund et al. 2011); Trine Hansen & Finn Kirpekar, unpublished data], but the responsible enzymes need to be identified in order to investigate the significance of the modifications. The focus of the present work is identification of the methyltransferase that adds a methyl group to Carbon-5 of C2499 in *D. radiodurans* 23S rRNA. Methylation of C2499 (position C2478 in true *D. radiodurans* numbering) has so far not been found in other organisms, even the relatively closely related *T. thermophilus* (Mengel-Jorgensen et al. 2006; Havelund et al. 2011), suggesting m^5^C2499 to be a species-distinct characteristic. C2499 is located in the peptidyltransferase centre of the ribosome (Fig. 1a), and C2499 has been proposed to form a wobble base pair with A2453 that is responsible for a pH-dependent conformational change of this catalytic centre in *E. coli* (Bayfield et al. 2004). C2499 has also been implicated in antibiotic resistance: It has been shown to form “third layer” interactions in the *D. radiodurans* 50S ribosomal subunit binding of a series translation-inhibiting antibiotics (Davidovich et al. 2008); additionally, a C2499-to-U mutation induces sparsomycin resistance in the archaeon *Halobacterium halobium* (Tan et al. 1996). Hence, several intriguing features can be attributed to C2499, and a delineation of the impact of its position-5 methylation is of significant interest.

Through gene cloning, in vitro and in vivo methylation studies and gene knockout, we show that the *D. radiodurans* open reading frame *DR_0049* (GenBank Gene ID: 1799079) encodes the methyltransferase that is responsible for the synthesis of m^5^C2499. The *DR_0049* knockout strain of *D. radiodurans* opens for a thorough investigation of the function of m^5^C2499.

## Materials and methods

*D. radiodurans* strain R1 was obtained from DSMZ (Brauschweig, Germany) and was grown at 30 °C on 10 g/L casein-based peptone, 5 g/L yeast extract, 5 g/L glucose, and 5 g/L NaCl (pH 7.3) with shaking in liquid culture; 16 g/L agar was added for growth on solid medium. Plasmids pKatCAT3 and pDR1089cat (Satoh et al. 2012) were used for *DR_0049* knockout constructs and control for *D. radiodurans* transformation.

### Cloning of *DR_0049*

*D.radiodurans* genomic DNA was purified using High Pure PCR Template Preparation Kit (Roche applied science) with cell lysis in the supplied tissue lysis buffer. The *D. radiodurans* open reading frame *DR_0049* was PCR amplified from genomic DNA: 50 µL PCR contained 120 ng DNA, 0.5 µM of each of the primers CTATCTTCGCCATATGTCCGC and CACCCTGAAAAAGATCTGTCCATC, 200 µM of each dNTP, 1 unit Phusion DNA polymerase (New England Biolabs), 6 % DMSO and 1× Phusion GC buffer. The PCR temperature cycling was: 98 °C/60 s; 30 × (98 °C/5 s, 57 °C/15 s, and 72 °C/45 s); and 72 °C/600 s. The desired PCR product was purified via agarose gel electrophoresis using the NucleoSpin Extract II kit (Macherey–Nagel) with AE buffer from NucleoSpin Plasmid Purification kit (Macherey–Nagel). The PCR fragment was digested with restriction enzymes NdeI and BglII (cleavage sites present in the PCR primers) and inserted into the expression vector pLJ102 as previously described (Andersen and Douthwaite 2006), generating an isopropyl-1-thio-*β*-*D*-galactopyranoside (IPTG)-inducible construct for the recombinant protein with a *C*-terminal histidine_6_ tag. This plasmid—named pLJ102-DR0049—was subsequently transformed into *E. coli* strain Top10 with selection for ampicillin resistance.

### Investigation of C2499 methylation in *E. coli*

200 mL LB medium was inoculated with 0.5 mL overnight culture of *E. coli* Top10 containing pLJ102-DR0049 and grown at 37 °C with shaking until OD_450_ = 0.45. The culture was placed on ice for 10 min, transferred to a JA14 tube and centrifuged at 6000 rpm for 10 min at 4 °C. The cell pellet was resuspended in 100 mL of cold TMN buffer (50 mM Tris–HCl (pH 7.5), 10 mM MgCl_2_, 100 mM NH_4_Cl), pelleted again by centrifugation and resuspended in 2 ml TMN buffer. The cells were lysed by seven sonications for 30 s with 30 s pause between each sonication. Cell debris was removed twice by a 16,000 rpm centrifugation for 10 min at 4 °C, decanting the supernatant to a new microtubes tube after each centrifugation. The RNA was extracted three times with phenol, one time with phenol/chloroform (1:1 mixture) and one time with chloroform followed by precipitation with sodium acetate (pH 6.5)/ethanol. A 23S rRNA subfragment encompassing nucleotides 2480–2527 was purified by hybridisation of the DNA oligonucleotide GCCCCAGGATGTGATGAGCCGACATCGAGGTGCCAAACACCGCCGTCG to total cellular RNA followed by degradation of single-stranded RNA and subfragment purification via polyacrylamide gel; details have been reported (Andersen et al. 2004). The position 2480–2527 23S rRNA subfragment was digested with RNase A and analysed by matrix-assisted laser desorption/ionisation (MALDI) mass spectrometry on a Bruker Ultraflextreme instrument in positive ion mode using 3-hydroxypicolinic acid as matrix (Douthwaite and Kirpekar 2007). Tandem mass spectrometry was performed on a Waters QTOF MALDI Premier instrument (Douthwaite and Kirpekar 2007).

### Investigation of DR0049 methylation activity in vitro

200 ml LB culture of *E. coli* Top10 containing pLJ102-DR0049 was grown to exponential phase at 37 °C with vigorous shaking. IPTG was added to a final concentration of 1 mM followed by incubation at 37 °C for 3 h. Cells were harvested and lysed by sonication as described above. Recombinant DR0049 containing the C-terminal Histidine_6_-tag was purified using a Ni–NTA agarose column (Qiagen) according to the manufacturer’s instructions. The eluted fractions containing DR0049—as identified by polyacrylamide gel electrophoresis—were dialysed at 4 °C against [20 mM Tris–HCl (pH 7.8), 10 mM MgCl_2_, 6 mM β-mercaptoethanol, 10 % glycerol, 250 mM NH_4_Cl] with two buffer replacement for a total of 24 h.

In vitro methylation was carried out on total *E. coli* RNA, purified as described above. 300 µl 2× in vitro methylation buffer (40 mM HEPES pH 7.5, 200 mM NH_4_Cl, 10 mM MgCl_2_, 20 % glycerol, 10 mM β-mercaptoethanol) and a volume of water that will yield a final 1× in vitro methylation buffer concentration were mixed and incubated at room temperature for 15 min. Total RNA containing around 200 pmol of 23S rRNA was added, and the mix was incubated at 50 °C for 5 min. The mix was cooled to 37 °C before adding 125 pmol of DR0049 and *S*-adenosylmethionine (SAM) to a final concentration of 0.5 mM in a total volume of 600 µl. The reaction was incubated at 37 °C for 1 h. RNA was recovered by phenol/chloroform extraction and sodium acetate/ethanol precipitation. Purification of the 23S rRNA subfragment and MALDI mass spectrometry was done as described above.

### Constructs for *D. radiodurans* gene knockout

A 5′ and 3′-end truncated version of *DR_0049* was PCR amplified from *D. radiodurans* genomic DNA: 50 µL PCR contained 50 ng DNA, 0.5 µM of each primer TGTACGAGTCGCATATGCGCGA and CAGAATCACGAGATCTTAGTCGGC, 200 µM of each dNTP, 1 unit Phusion DNA polymerase (New England Biolabs), 6 % DMSO and 1× Phusion GC buffer. The PCR temperature cycling was as follows: 98 °C/60 s; 30× (98 °C/5 s, 58 °C/15 s and 72 °C/45 s); and 72 °C/600 s. The PCR product was purified and digested with BglII/NdeI, which generates a version of *DR_0049* lacking nucleotides 1-119 and 927-1227. This DNA fragment was cloned into BamHI/NdeI cleaved pUC18 to generate the construct pDR0049 that was propagated in *E. coli* strain Top10.

A chloramphenicol acetyltransferase (*cat*) gene with a promoter functional in *D. radiodurans* was obtained from the plasmid pDR1089cat (Satoh et al. 2012): 300 ng of plasmid template were mixed with 0.5 µM of primers GCCAGGGTTACCCCAGTC and GCTCGGTAACCGGGGAT, 200 µM of each dNTP, 1 unit Phusion DNA polymerase (New England Biolabs) and 1× Phusion GC buffer and subjected to a PCR temperature program of 98 °C/60 s; 30× (98 °C/5 s, 58 °C/15 s, and 72 °C/45 s); and 72 °C/600 s. The PCR primers introduced BstEII restriction enzyme sites into the PCR product, which were used to insert it into pDR0049 to obtain pDR0049::cat; the BstEII site is located at position 235 in the truncated version of *DR_0049*, which is hence interrupted by the *cat* gene. Restriction enzyme analysis revealed that the *cat* gene was inserted in the same transcriptional direction as *DR_0049*.

*D. radiodurans* was transformed with pDR0049::cat (and pDR1089cat as positive control for transformation) as follows: 25 ml *D. radiodurans* culture was grown at 30 °C with shaking in TGYN broth (10 g/L tryptone, 5.0 g/L yeast extract 5 g/L glucose, 5 g/L NaCl) to an OD_600_ of 0.7, harvested by centrifugation (6000 rpm, 4 °C, 10 min in an Eppendorf 5810R centrifuge) and resuspended in 2 ml TGYN broth containing 30 mM CaCl_2_. 1 µg DNA in 10 µl water was added to 100 µl cells followed by incubations on ice for 15 min and 30 °C for 90 min. 4 ml TGYN broth was added and cellular growth was done by incubating at 30 °C for 16 h with gently shaking. 1 ml cells were harvested by centrifugation at 6000 rpm in a micro centrifuge, resuspended in 100 µl TGYN and plated on TGYN plates containing 3 µg/ml chloramphenicol. Plates were inspected for chloramphenicol-resistant colonies after 3–6 days of incubation at 30 °C. To investigate if and how pDR0049::cat was integrated into the genomic *DR_0049* locus, genomic DNA from chloramphenicol-resistant colonies was subjected to PCR using the primers GAGGGGAGACTAAAACTCCA and CACCCTGAAAAATATCAGTCCAT, which anneal just upstream and downstream of the *DR_0049* open reading frame, respectively. The PCR product was inspected by agarose gel electrophoresis using a similarly generated PCR product from wild-type *D. radiodurans* as control.

### Investigation of C2499 methylation in *D. radiodurans*

*D. radiodurans* was grown in TGY broth at 30 °C with shaking for 3 days and harvested by centrifugation at 6000 rpm and 4 °C for 15 min in an Eppendorf 5810R centrifuge. The supernatant was removed, pellets placed on ice and resuspended in 900μL TriReagent (Sigma-Aldrich). Cells were lysed on a FastPrep FP120 Cell Disrupter instrument (ThermoSavant) with three runs at speed 6 for 40 s; the samples placed on ice between each run. The RNA was extracted with chloroform twice and precipitated with ethanol and sodium acetate (pH 4.5). The RNA was pelleted by centrifugation at max speed at 4 °C for 30 min in a micro centrifuge and washed in 70 % ethanol. The air-dried pellet was resuspended in 50μL H_2_O. A 47 nucleotide 23S rRNA subfragment was purified and analysed by mass spectrometry as described above, except that the DNA oligonucleotide GCCCCAGGATGCGACGAGCCGACATCGAGGTGCCAAACCTCCCCGCC was used for hybridisation.

## Results

The 23S rRNA of *D. radiodurans* is posttranscriptionally modified with m^5^C at position 1942 and 2499 ((Havelund et al. 2011); Trine Hansen and Finn Kirpekar, unpublished data). The related bacterium *T. thermophilus* only harbours an m^5^C1942 with the modifying enzyme identified and dubbed RlmO (Larsen et al. 2012). *T. thermophilus* RlmO was used as query in a Blast search against all *D. radiodurans* (strain R1) translated open reading frames in order to uncover candidates for rRNA m^5^C methyltransferases. The two highest scoring hypothetical proteins were encoded by *DR_1694* and *DR_0049* with *Expect values* of 2 × 10^−89^ and 10^−40^, respectively. The two corresponding genes were successfully PCR amplified from *D. radiodurans* genomic DNA, but despite several attempts only the *DR_0049* gene could be cloned into an expression vector and propagated in *E. coli*.

Because DR1694 has the greatest similarity with *T. thermophilus* RlmO, our a priori hypothesis was that DR0049 is responsible for methylation of C2499. C2499 is unmodified in *E.coli*, so the introduction of the *DR_0049* gene in an expression vector may result in 23S rRNA m^5^C2499 synthesis, if our hypothesis was correct. Isolation of a 23S rRNA subfragment encompassing C2499 followed by RNase A digestion, and mass spectrometric analysis revealed a signal corresponding to A_2497_CmC_2499_-p plus an extra methyl group (Fig. 1b) compared with an *E. coli* control not containing the *DR_0049* gene (Cm2498 is a well characterised 23S rRNA modification in *E. coli* (Kowalak et al. 1995)). The location of the additional methyl group to the nucleobase of C2499 was substantiated by tandem mass spectrometry (Fig. 1c). We additionally performed in vitro methylation of *E. coli* 23S rRNA with recombinantly expressed DR0049 with essentially same outcome by both mass spectrometric and tandem mass spectrometric analyses as the in vivo methylation data (not shown). Hence, DR0049 is functional in *E. coli* methylating 23S rRNA at position C2499 both in vivo and in vitro, while no signs of C1942 methylation were observed (supplementary Fig. 1). It should be noted that the *E. coli* strain expressing DR0049 did not show any obvious growth deficiencies under the conditions used in this work.

Definitive proof of the function of DR0049 requires investigations in *D. radiodurans*. We wanted to inactivate the genomic *DR_0049* gene through insertion of a chloramphenicol resistance cassette by homologous recombination. Earlier works reported both double (Funayama et al. 1999; Satoh et al. 2012) and single (Smith et al. 1988) homologous cross-over events as routes for efficient recombination between plasmid DNA harbouring an interrupted version of the gene of interest and the genomic copy of the gene. Double homologous recombination will result in plain insertion of the chloramphenicol resistance cassette and therefore gene disruption in antibiotic-resistant clones. A single-homologous recombination between a plasmid-borne, chloramphenicol resistance-interrupted *DR_0049* gene and the genomic *DR_0049* will, however, result in one intact and one interrupted *DR_0049* gene in the genome. In analogy with an earlier approach (Markillie et al. 1999), the plasmid-borne *DR_0049* gene was therefore truncated in both the 5′ and the 3′ end of the coding region (plasmid pDR0049::cat; see “Materials and methods” for details) to ensure the absence of functional *DR_0049* if the single recombination event governed in our experiments.

**Fig. 2:**
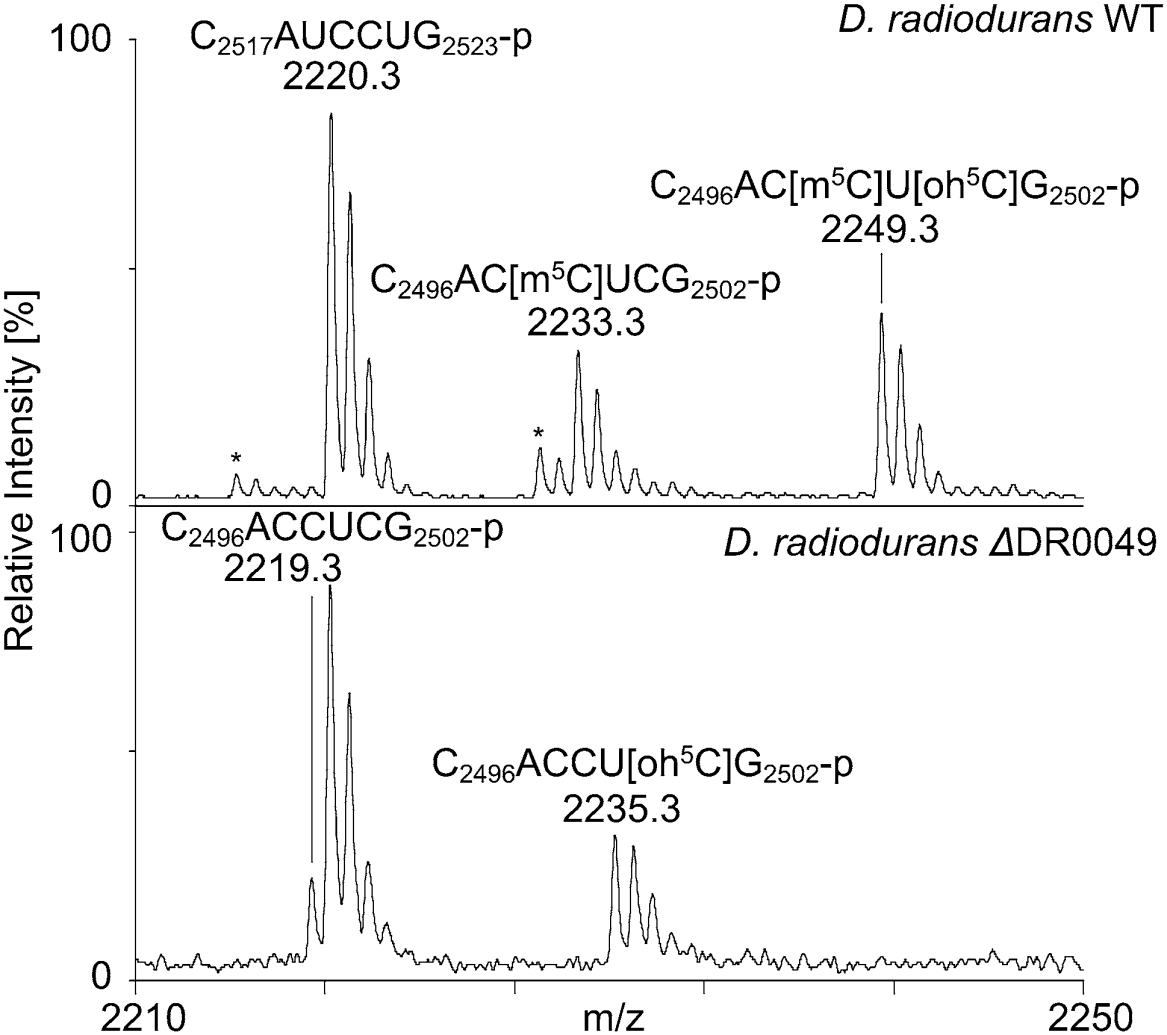
C2499 methylation of 23S rRNA depends on *DR_0049* in *D. radiodurans* as determined by mass spectrometry. RNase T1 generated products encompassing C2499 display a 14.0 Da. mass difference between the WT and the ΔDR0049 strains of *D. radiodurans*; the *m*/*z* 2220.3 signals arise from the C_2517_AUCCUG_2523_-p product and remain unaltered between the two strains. Asterisk-labelled signals correspond to minor RNase T1 products with a 2′-3′-cyclic phosphate

Transformation of *D. radiodurans* with pDR0049::cat yielded few chloramphenicol-resistant colonies that were visible only after incubation for 6 days. Transformation with a “positive” control, pDR1089cat (Satoh et al. 2012) harbouring an interrupted *recF* gene, generated hundreds of colonies after a couple of days, which suggests that disruption of *DR_0049* has a severe influence on *D. radiodurans* viability and general fitness. Initial verification of the *DR_0049* gene disruption was done by PCR with primers that flanked the coding region of the gene. The PCR product was larger by 1 kbp, which equals the size of the *cat* gene, than the product from the wild-type *D. radiodurans* (supplementary Fig. 2). This reveals that the *cat* gene was inserted into *DR_0049* by a double recombination event, one in each of the *DR_0049* portions flanking the *cat* gene. When analysing the modification status of C2499 in 23S rRNA of *D. radiodurans* with the interrupted *DR_0049* gene (strain called ∆DR0049 henceforth) by MALDI mass spectrometry, we observed total absence of the methylation (Fig. 2). A signal at *m*/*z* 2235.3 in ∆DR0049 corresponds to C_2496_ACCU[oh^5^C]G_2502_-p with the 5-hydroxylation of C2501 in accordance with our previous report (Havelund et al. 2011). A minor signal at *m*/*z* 2219.3 is an unmodified version of C_2496_ACCUCG_2502_-p; hence the oh^5^C2501 is only a partial modification in these experiments. The wild type displays the same signals, but with an offset of plus 14.0 Da, which leads to the conclusion that *DR_0049* encodes the methyltransferase that adds a methyl group to Carbon-5 of C2499 in *D. radiodurans* 23S rRNA.

The low transformation efficiency of the *DR_0049::cat* construct together with the slow appearance of visible colonies prompted us to look closer at the *D. radiodurans* ∆DR0049 growth rate in an established culture. The wild type had an exponential phase-doubling time of around 2½ hours at standard growth conditions and reached a stationary phase OD_600_ of ~2 within 12–14 h. The ∆DR0049 strain required around 5 days to obtain a stationary phase OD_600_ of ~0.5, and the slow growth rate precluded reliable determination of doubling time. Thus, these preliminary studies reveal a severe growth defect associated with inactivation of the *DR_0049* gene.

## Discussion

The delineation of DR0049 as the m^5^C2499-forming enzyme opens for studies on the significance of this 23S rRNA modification. Already the construction of the ∆DR0049 strain indicated that we are dealing with an rRNA modification that is crucial for the bacterium as transformants appeared at a low frequency and much slower than a control transformation with a *DR_1089::cat* construct. The requirement of the *DR_0049* gene/gene product persisted in the established culture of the ∆DR0049 strain. The same chloramphenicol resistance cassette was used in both cases, thus the inactivation of *DR_0049* versus *DR_1089* is likely the reason for the difference. It is also possible that insertion of the actively transcribed chloramphenicol resistance cassette has an impact on the expression of nearby genes, but we do not favour this explanation, since *DR_0049* is the last gene in a cluster/putative operon with all members having the same transcriptional direction (and the same direction as the inserted chloramphenicol resistance gene).

**Fig. 3:**
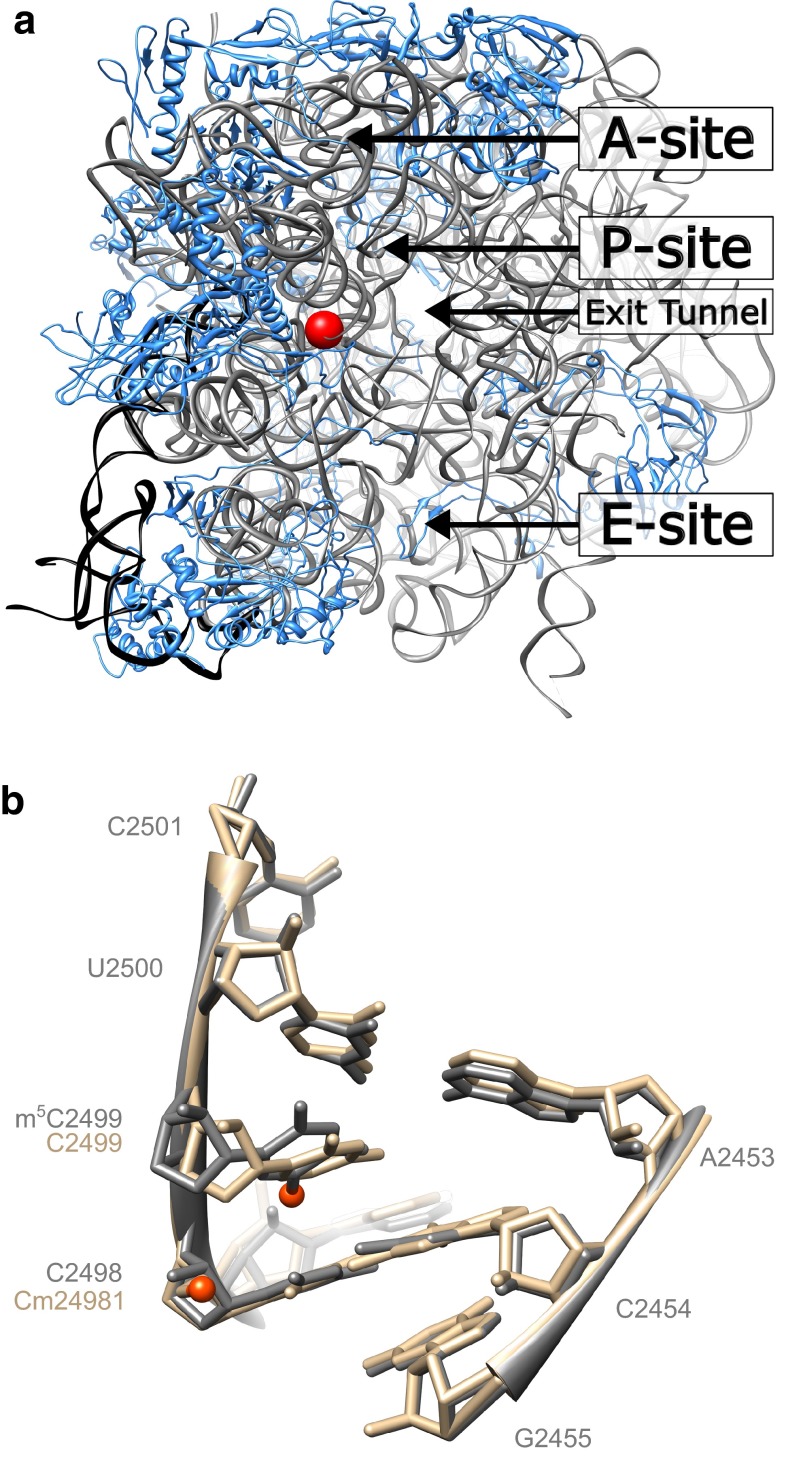
C2499 in the context of the 50S ribosomal subunit. **a** Side view of the *D. radiodurans* 50S ribosomal subunit looking up through the exit-tunnel from the interface to the 30S subunit. m^5^C2499 is emphasised by the *red sphere* and central features of the 50S subunit are indicated. *Grey ribbon* is 23S rRNA, *black ribbon* is 5S rRNA and *blue structures* are ribosomal proteins. Modified from PDB file 2ZJR (Harms et al. 2008). **b** Overlay of the C2499 surroundings of *D. radiodurans* (in *grey*) and *E. coli* (in beige) with nucleotide methylations highlighted by orange spheres. Modified from PDB files 2ZJR and 4YBB (Noeske et al. 2015), respectively

The m^5^C2499 modification is so far only identified in *D. radiodurans* 23S rRNA, where it is located very much in the interior of the 50S subunit (Fig. 3a). The traditional secondary structure of bacterial 23S rRNA depicts positions A2497 to U2506 as part of the peptidyltrasferase loop and not engaged in base pairing (Fig. 1a). However, the X-ray crystallography structure of the *D. radiodurans* 50S ribosomal subunit reveals an extension of helix 89 where C2498 base pairs with G2454, and U2500 forms a pair with A2453 while m^5^C2499 inserts between its two neighbouring bases distorting the A-helix structure (Fig. 3b). C2499 is tilted so dispersion force interactions between its methyl group and the nucleobase of C2498 can occur; the similar region of *E. coli* 23S rRNA has a nearly identical structure except that the C2499 nucleobase is more planar with its neighbours. From these structural data, it is by no means easy to account for a well-defined function of the C2499 methylation or for the apparent severity of its absence. The PDB 2ZJR and 4YBB structures that form the basis for Fig. 3b are incompatible with the previously suggested A2453-C2499 wobble base pair (Bayfield et al. 2004), but it cannot be excluded that this base pair and the modification are of relevance during protein synthesis, since a ribosome structure is only a snapshot of the translation process. However, we favour a function of DR0049 in ribosome assembly as has been indicated for other rRNA-modifying enzymes (Raychaudhuri et al. 1998; Bügl et al. 2000; Caldas et al. 2000; Gutgsell et al. 2005), which may be investigated by e.g. non-equilibrium sucrose gradient centrifugation.

A Blast search suggests that the m^5^C2499 modification is likely to be present in other *Deinococcus* species, since these contain open reading frames for highly similar proteins, but also species belong to numerous other bacterial phyla harbour such genes. Notably, *Thermus* species do not encode such highly similar proteins in agreement with the absence of a C2499 methylation in *T. thermophilus*. It will hence be relevant to investigate the correlation between m^5^C2499 and tolerance to various stress conditions in some of the above bacteria to obtain an idea about this methylation’s contribution to the characteristic phenotype of *D. radiodurans*.

## Electronic supplementary material

Supplementary material 1 (DOCX 299 kb)

## Abbreviations

Cat: Chloramphenicol acetyltransferase
Cm: 2′-*O*-methylcytidine
DMSO: Dimethyl sulfoxide
dNTP: 2′-deoxynucleoside triphosphate
HEPES: 4-(2-hydroxyethyl)-1-piperazineethanesulfonic acid
IPTG: Isopropyl-1-thio-*β*-*D*-galactopyranoside
kbp: Kilo basepairs
LB: Luria-Bertani
*N*^2^,*N*^2^-dimethylguanosine
m^5^C: 5-methylcytidine
*m*/*z*: Mass-to-charge ratio
MALDI: Matrix-assisted laser desorption/ionisation
Ni-NTA: Nickel-nitrilotriacetic acid
oh^5^C: 5-hydroxycytidine
-p: 3′-terminal phosphate
>p: 2′–3′-terminal cyclic phosphate
PCR: Polymerase chain reaction
rRNA: Ribosomal RNA
SAM: *S*-adenosylmethionine
tRNA: Transfer RNA
Um: 2′-*O*-methyluridine
WT: Wild type
