# Supplementary material for: Identification of the methyltransferase targeting C2499 in Deinococcus radiodurans 23S ribosomal RNA
Source: Extremophiles. 2015 Nov 21;20:91–9. doi: 10.1007/s00792-015-0800-z (PMC4690841; doi:10.1007/s00792-015-0800-z)
Supplement: Supplementary file 1 — Supplementary material 1 (DOCX 299 kb) [file 792_2015_800_MOESM1_ESM.docx]

**Supplemetary material**

**Identification of the methyltransferase targeting C2499 in *Deinococcus radiodurans* 23S ribosomal RNA**

Julie Mundus, Karen Freund Flyvbjerg & Finn Kirpekar*


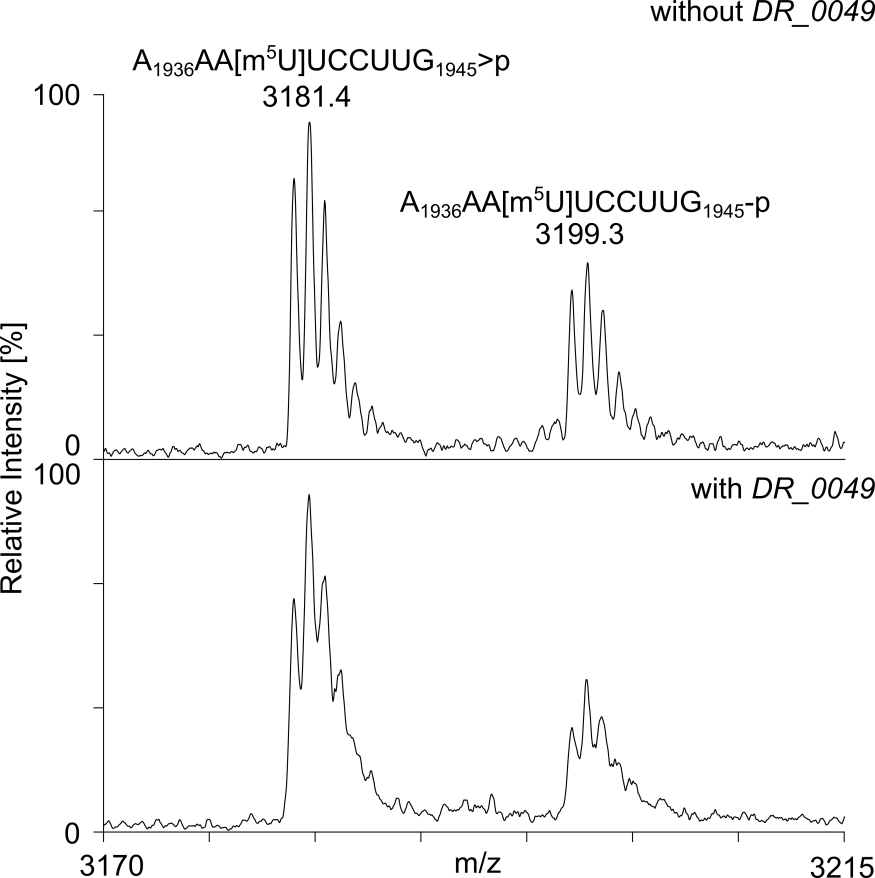


Supplementary figure 1. *E. coli* C1942 of 23S rRNA is not modified by DR0049.

Mass spectrometric analysis of the RNase T1 generated product A_1936_AA[m^5^U]UCCUUG_1945_ of 23S rRNA. Upper panel shows signal from the original Top10 *E. coli* strain while the lower panel displays the signals from *E. coli* Top10 with an expressed *DR_0049* gene. Both the 2´-3´-cyclic phosphate and the 3´-phosphate products of RNase T1 digestion are present.


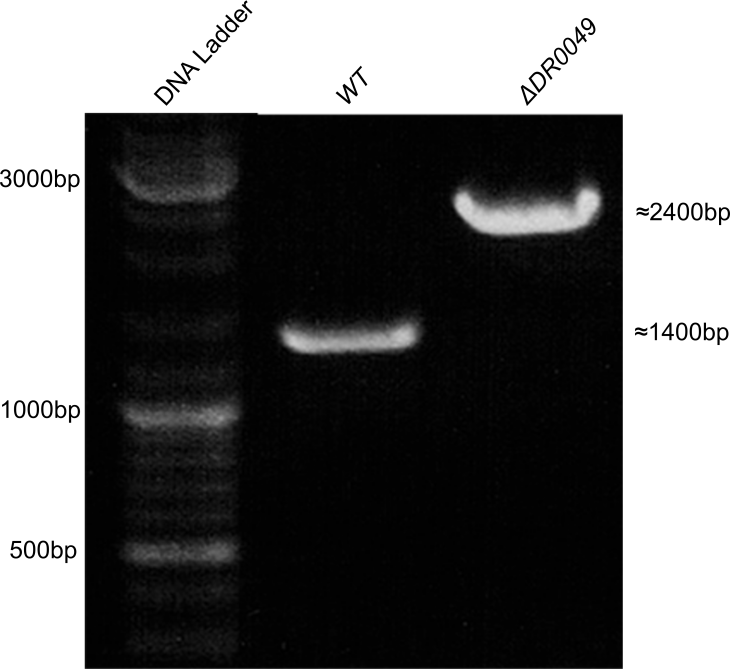


Supplementary figure 2. PCR verification of *cat* gene insertion into *DR_0049*.

Primers flanking the *DR_0049* gene were used for PCR analysis of the WT and theΔDR0049 *D. radiodurans* strains (lanes 2 and 3, respectively). The ΔDR0049 strain gives a product that is increased by the number of base pairs in the inserted *cat* gene compared to the WT. Lane 1 is a DNA size standard ladder.
